# Reply to van der Pluijm et al. Comment on “Weitzman et al. Resistance to Antimalarial Monotherapy Is Cyclic. *J. Clin. Med.* 2022, *11*, 781”

**DOI:** 10.3390/jcm11112972

**Published:** 2022-05-25

**Authors:** Ortal Calfon-Peretz, Rachel Weitzman, Moshe Amitay, Abraham O. Samson

**Affiliations:** 1 Bioinformatic Department, Jerusalem College of Technology, Jerusalem 9372115, Israel; w.rachel99@gmail.com (R.W.); mosh9900@gmail.com (M.A.); 2 Drug Discovery Lab, Azrieli Faculty of Medicine, Bar Ilan University, Safed 1311502, Israel; avraham.samson@biu.ac.il

Thank you for the opportunity to respond to comments gracefully raised by van der Pluijm et al. [1] and referring to our paper titled: “Resistance to antimalarial monotherapy is cyclic” [2]. In this paper, we used PubMed text mining to determine the trends of antimalarial drug resistance over the last four decades. First, we counted the number of citations containing “drug” AND “malaria” AND “resistance”. Then, we normalize this count and divide by the number of citations containing “drug” OR “malaria” OR “resistance”. The normalized count was then used as an indicator of malaria drug resistance, for each year, using the equation below (Equation (1)). Notably, the normalized citations rise and fall in a cyclic manner, which is similar to experimental and clinical resistance reported by the Worldwide Antimalarial Resistance Network (WWARN), among others.

(1) $$Normalized\;citation=\frac{ [drug\;AND\;malaria\;AND\;resistance]}{ [drug\;OR\;malaria\;OR\;resistance]}$$

First, as a potential limitation to our study, van der Pluijm et al. correctly state that the nominator of this equation is “polluted” by publications that randomly happen to mention the drug of interest and the word “resistance”. To offset this “pollution”, we included only drugs with more than 20 yearly citations. Assuming random noise, we ignored any drug with signal-to-noise ratios below 4.67 (S = 20, N = $\sqrt{20}$). Furthermore, to reduce random noise, we applied a 3-year average smoothing function on the normalized citations. Finally, to further reduce the chance of random co-occurrence, we queried only the title, abstract, and other terms of PubMed citations, but not the full text paper. These measures significantly reduced random pollution, and we do not claim that noise is completely eliminated.

Second, as a potential limitation to our study, van der Pluijm et al. correctly state that both known and unknown factors contribute to the rise and fall of normalized citations. For example, Halofantrine was first marketed in 1984, and not surprisingly, its citations only begin in that year. Notably, the citations decline after 1995, following concerns over halofantrine cardiotoxicity. We agree that resistance dissipates with infrequent use and adheres to Darwin’s principle of natural selection. Thus, given the right conditions, resistance can fall after linear rises. For example, we hypothesize that artemisinin resistance can decrease following a period of discontinued use. We do not know if rising artemisinin resistance evolved in ancient China or whether it predates modern medicine. Our data suggest that artemisinin resistance was registered before 2008–2009 and evolved gradually. We know that, given natural selection, ancient genes gradually evolve new functions, such as drug resistance, and that new functions gradually degenerate without natural selection. This is clearly illustrated in the example brought forward by van der Pluijm et al. in Sub-Saharan Africa, where chloroquine resistance has declined after a period of infrequent use. We believe that antimalarial resistance is governed mostly by its use, very much like antibiotic drug resistance [3]. We agree that different artemisinins share similar modes of action and that their active metabolite is dihydroartemisinin. However, unlike other artemisinins, we show that artesunate antimalarial resistance citations have declined since 2006, but we do not know why.

Finally, as a potential limitation to our study, van der Pluijm et al. correctly state that normalized citations cannot accurately predict future trends. However, given enough data, we believe that resistance trends may be approximately projected.
